# Supplementary material for: Associations Between Nutrition, Gut Microbiome, and Health in A Novel Nonhuman Primate Model
Source: Sci Rep. 2018 Jul 24;8:11159. doi: 10.1038/s41598-018-29277-x (PMC6057944; doi:10.1038/s41598-018-29277-x)
Supplement: Supplementary file 1 — Dataset 1 [file 41598_2018_29277_MOESM1_ESM.doc]

**Supplementary Data for “Associations Between Nutrition, Gut Microbiome, and Health in A Novel Nonhuman Primate Model”**

Jonathan B. Clayton^a,b,c^, Gabriel A. Al-Ghalith^d^, Ha Thang Long^b,c,e^, Bui Van Tuan^b^, Francis Cabana^c,f^, Hu Huang^d^, Pajau Vangay^d,g^, Tonya Ward^g^, Vo Van Minh^h^, Nguyen Ai Tam^b^, Nguyen Tat Dat^b^, Dominic A. Travis^c,i^, Michael P. Murtaugh^a^, Herbert Covert^j^, Kenneth E. Glander^c,k^, Tilo Nadler^l^, Barbara Toddes^m^, John C.M. Sha^n^, Randy Singer^a^, Dan Knights^c,d,g,o^, and Timothy J. Johnson^a,c,p*^

^a^Department of Veterinary and Biomedical Sciences, University of Minnesota, 1971 Commonwealth Avenue, Saint Paul, MN 55108, USA

^b^GreenViet Biodiversity Conservation Center, K39/21 Thanh Vinh Street, Son Tra District, Da Nang, Vietnam

^c^Primate Microbiome Project, 6-124 MCB, 420 Washington Ave SE, Minneapolis, MN 55455, USA

^d^Biomedical Informatics and Computational Biology, 200 Union St SE, University of Minnesota, Minneapolis, MN 55455, USA

^e^Frankfurt Zoological Society, Bernhard-Grzimek-Allee 1, 60316 Frankfurt, Germany

^f^Wildlife Nutrition Centre, Wildlife Reserves Singapore, 80 Mandai Lake Road, 729826, Singapore

^g^Biotechnology Institute, University of Minnesota, 1479 Gortner Avenue, Saint Paul, MN 55108, USA

^h^Faculty of Biology and Environmental Science, The University of Da Nang - University of Science and Education, 459 Ton Duc Thang Street, Lien Chieu District, Da Nang, Vietnam

^i^Department of Veterinary Population Medicine, University of Minnesota, 1365 Gortner Avenue, 225 Veterinary Medical Center, Saint Paul, MN 55108, USA

^j^Department of Anthropology, University of Colorado Boulder, 1350 Pleasant St, 233 UCB, Boulder, CO 80309, USA

^k^Department of Evolutionary Anthropology, Duke University, 130 Science Drive, 104 Biological Sciences, Durham, NC 27708, USA

^l^Endangered Primate Rescue Center, Cuc Phuong National Park, Nho Quan District, Ninh Binh Province, Vietnam

^m^Philadelphia Zoological Garden, 3400 West Girard Avenue, Philadelphia, PA 19108, USA

^n^School of Sociology and Anthropology, Sun Yat-Sen University, Guangzhou, 510275, China

^o^Department of Computer Science and Engineering, University of Minnesota, 4-192 Keller Hall, 200 Union St SE, Minneapolis, MN 55455, USA

^p^University of Minnesota, Mid-Central Research and Outreach Center, Willmar, Minnesota, USA

**SUPPLEMENTAL FIGURES LEGENDS:**


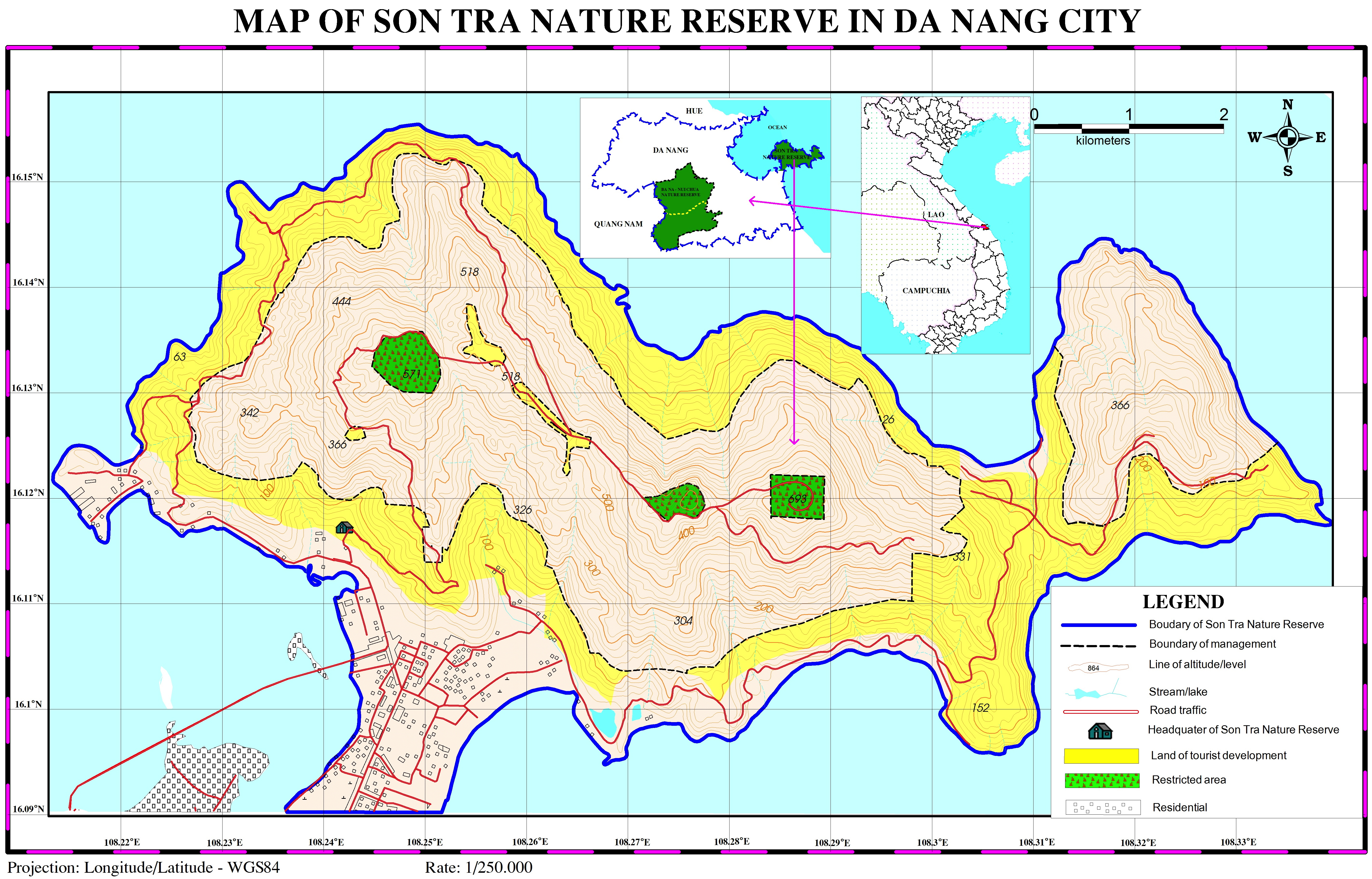


**Supplemental Figure 1.** **Study site**. Red-shanked doucs (*Pygathrix nemaeus*) inhabiting Son Tra Nature Reserve, Da Nang, Vietnam (16°06’—16°09’N, 108°13’—108°21’E) served as the wild population for this comparative study ^32,33^. Son Tra is located only 10 km from the heart of Da Nang City, which is the third largest city in Vietnam. The nature reserve is comprised of 4,439 total ha and of those 4,190 ha is covered by both primary and secondary forests ^32^. Our study area was approximately 600 ha and is located on the north central region of the peninsula.


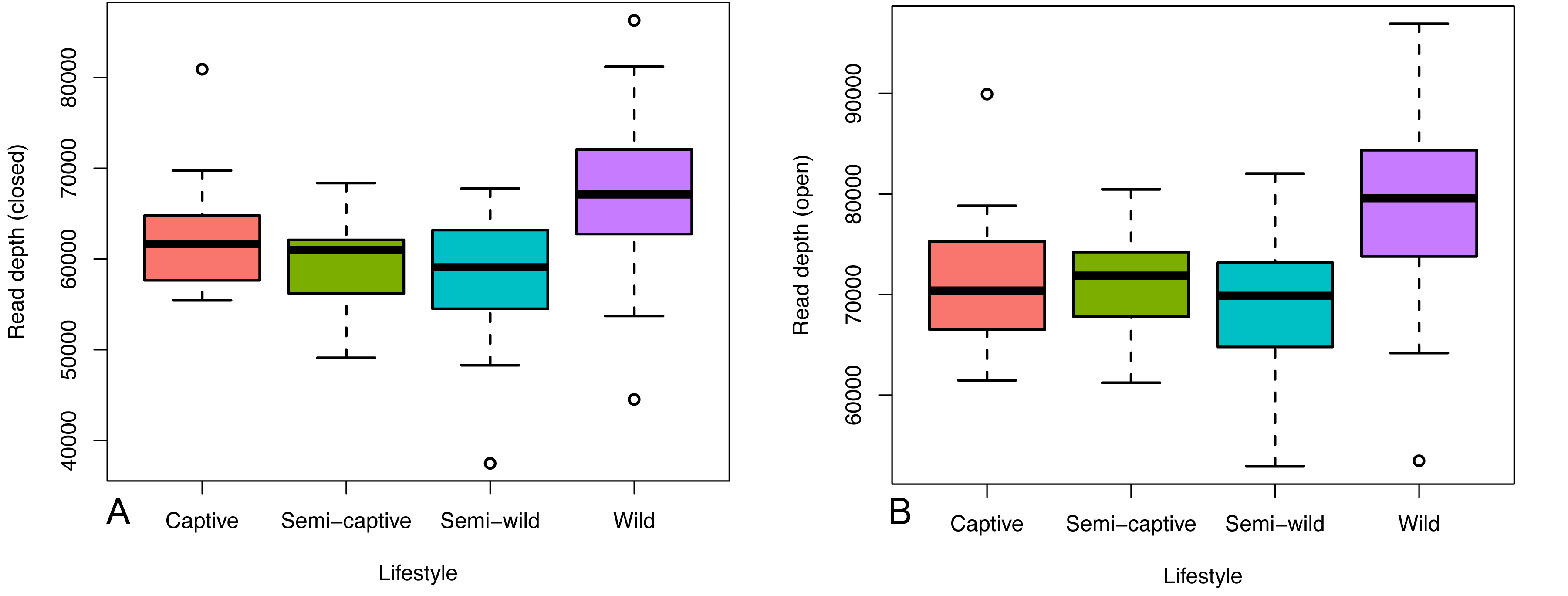


**Supplemental Figure 2. Read depth following quality control and OTU picking in the (a) closed-reference and (b) open-reference protocols.** Read depth was relatively uniform across lifestyles in both protocols and highest in the wild population. However, because the depth was statistically significantly different between the wild group and the others, we rarefied the data to even depth before analysis as mentioned in the Methods.


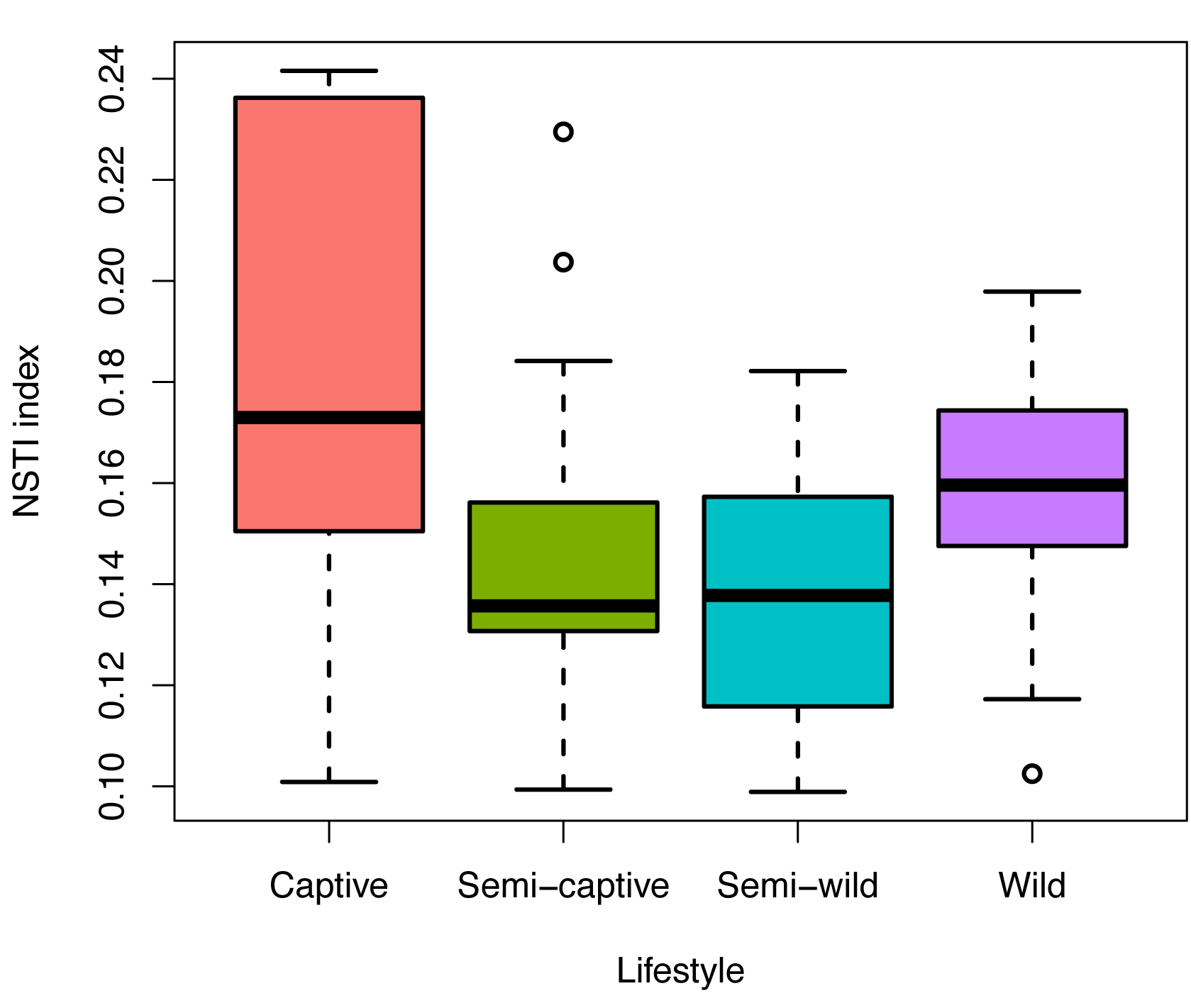


**Supplemental Figure 3. The Nearest Sequenced Taxon Index (NSTI) reported by PICRUSt for the lifestyle groups.** The mean NSTI index for all groups was below 0.18, and below 0.165 for all lifestyles except captive. A lower NSTI index means less phylogenetic distance between the reference genomes used for functional predictions. A value of 0.17 was reported ^52^ to be a “medium” level of distance, indicating reasonable reliability for the PICRUSt functional predictions. Interestingly, the captive group showed the highest overall NSTI scores, potentially indicating a smaller proportion of genomic content in this lifestyle group has been fully sequenced.

**Supplemental Figure 4.** **Diminished alpha diversity trends in red-shanked douc microbiomes across lifestyles are robust to collapsing by individual.** Violin plots of gut microbial alpha diversity across the 4 lifestyles according to (a) the number of species-like operational taxonomic units (OTUs) generated by open-reference OTU picking in the gut microbiome, and (b) the Shannon diversity index. The width of the shape corresponds to the distribution of samples (strips overlaid as strip chart), and asterisks denote significant differences at Welch’s t-test *p* < 0.05 (*), *p* < 0.01 (**), and *p* < 0.001 (***). Under both metrics, the wild population exhibits the highest biodiversity, which appears to diminish as a gradient with level of captivity to the captive population, which has the lowest. These trends are robust to collapsing samples by individual (captive n=2, semi-captive n=7, semi-wild n=18, wild n=9).


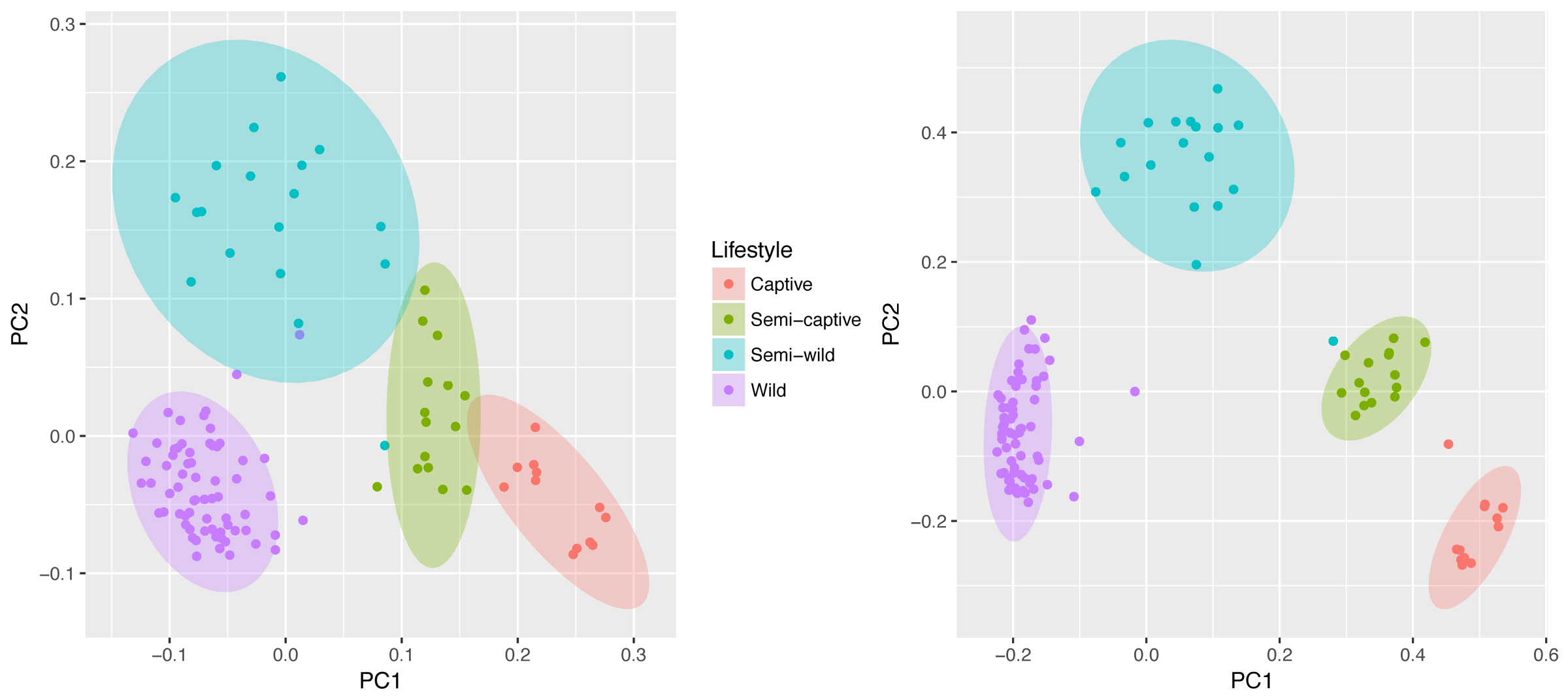


**Supplemental Figure 5. Principal coordinates plot of (a) weighted UniFrac and (b) Bray-Curtis metrics showing ecological distance between gut microbial communities in wild, semi-wild, semi-captive, and captive red-shanked doucs.** All samples were obtained with the same protocol for V4 16S rRNA sequencing, and open-reference OTU picking was used. Douc microbiomes clearly clustered by population suggesting that each douc population had a unique microbiome, and thus were highly distinctive from one another.


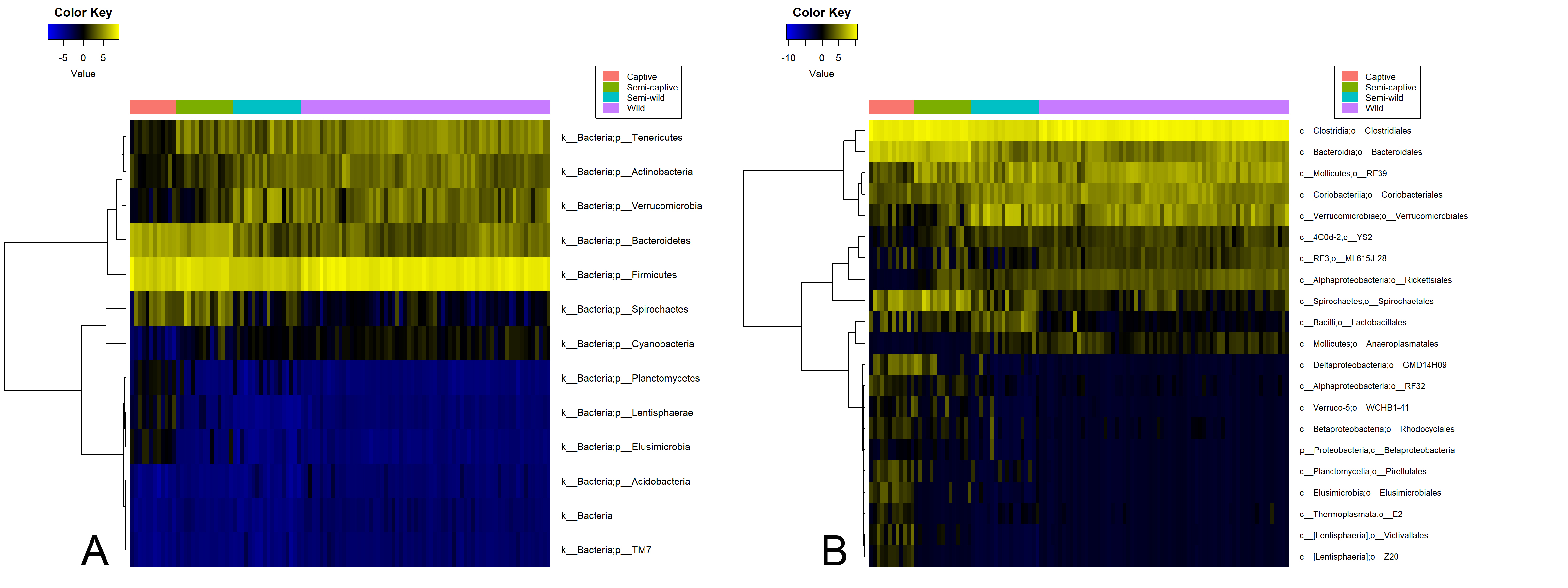


**Supplemental Figure 6. Heatmaps of differentially abundant microbial taxa at the phylum and order levels in red-shanked doucs living four distinct lifestyles.** Taxa are displayed with polyserial correlations (rho) above 0.3, rho estimate adjusted p < 0.05, and (pairwise) Wilcoxon rank-sum FDR-adjusted p-value comparing combined wild & semi-wild versus captive lifestyles < 0.05. Color represents intensity of centered log ratio abundances along gradient of color scale shown.


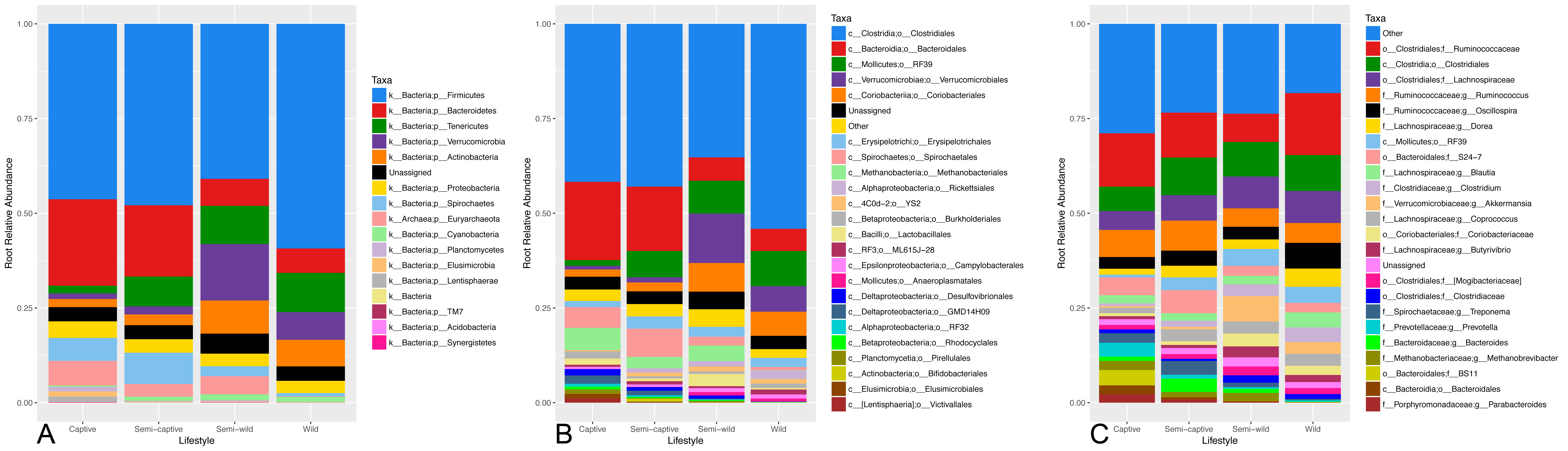


**Supplemental Figure 7.** **Stacked bar plots of microbial taxa relative abundance ordered by taxonomic level [(a) phylum, (b) order, and (c) genus] in wild, semi-wild, semi-captive, and captive red-shanked doucs**. All samples were obtained with the same open-reference V4 16S rRNA protocol. Up to 25 taxa (including a group for “Other”) are displayed. Taxa were prioritized for display according to highest groupwise maximum abundance, and ordered for display by average abundance throughout the dataset from top to bottom. For display purposes, the square root transformation of the abundance is shown rather than the absolute relative abundance to allow for easier visual inspection and comparison of lower-abundance community members. Legends show the known taxonomic rank of each taxon in the graph; colors may not be consistent between graphs. Microbes that could not be classified to the specified level are also included at the highest level at which they could be classified.

**Supplemental Figure 8.** **Taxonomic trends are robust to collapsing samples by individual.** Microbial taxa relative abundance ordered by taxonomic level [(a) phylum, (b) order, and (c) genus] in wild, semi-wild, semi-captive, and captive red-shanked doucs.


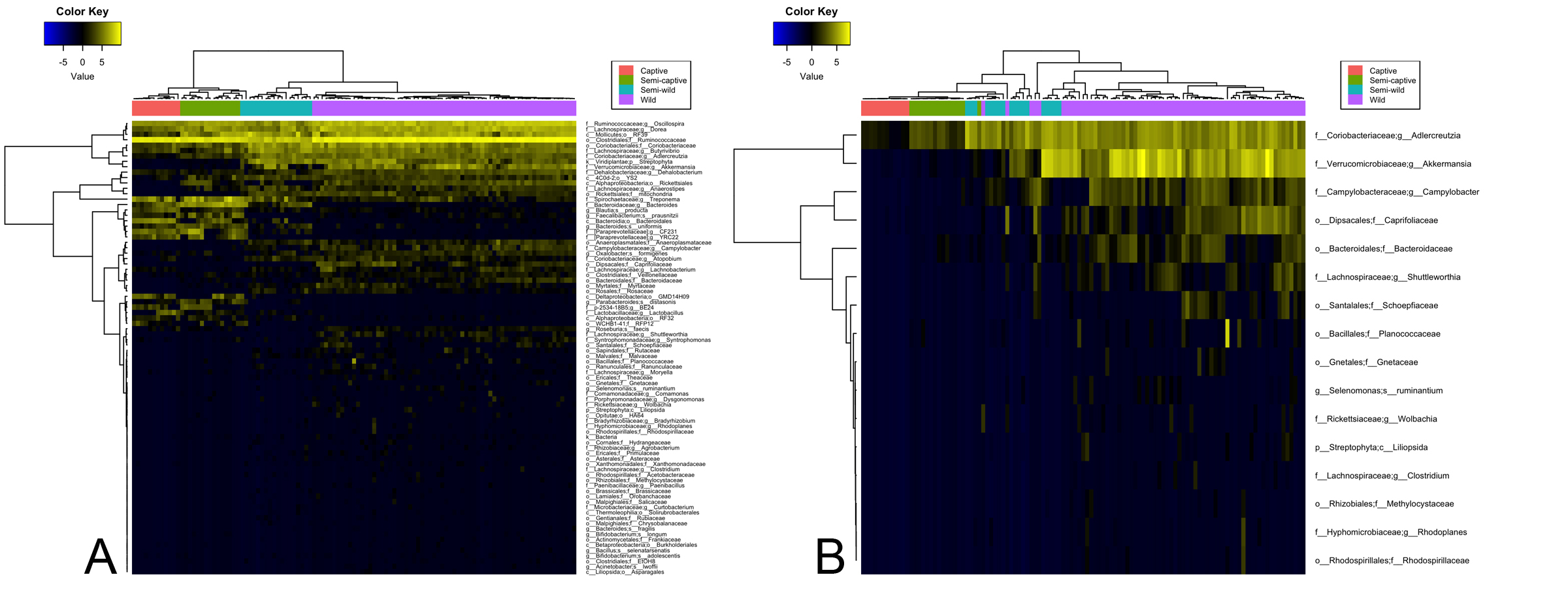


**Supplemental Figure 9. Biclustering of significantly differentiated taxa recapitulates group separation by lifestyle. (a)** A heatmap was generated using all of the statistically significant finest-grain features available in the data (up to the order level in plants and species level in prokaryotes). All features with polyserial rho > 0.6 and captive-wild adjusted Wilcoxon p < 0.05 were included. The heatmap was subjected to unsupervised complete linkage clustering of correlation dissimilarity [1 - abs(cor())], revealing that the correlation patterns of top differentiated taxa alone is sufficient to completely recover lifestyle group membership of the samples. This highlights the potential utility of taxa as biomarkers in this population. (b) A similar recovery is observed when selecting only the top 16 differential taxa for inclusion.


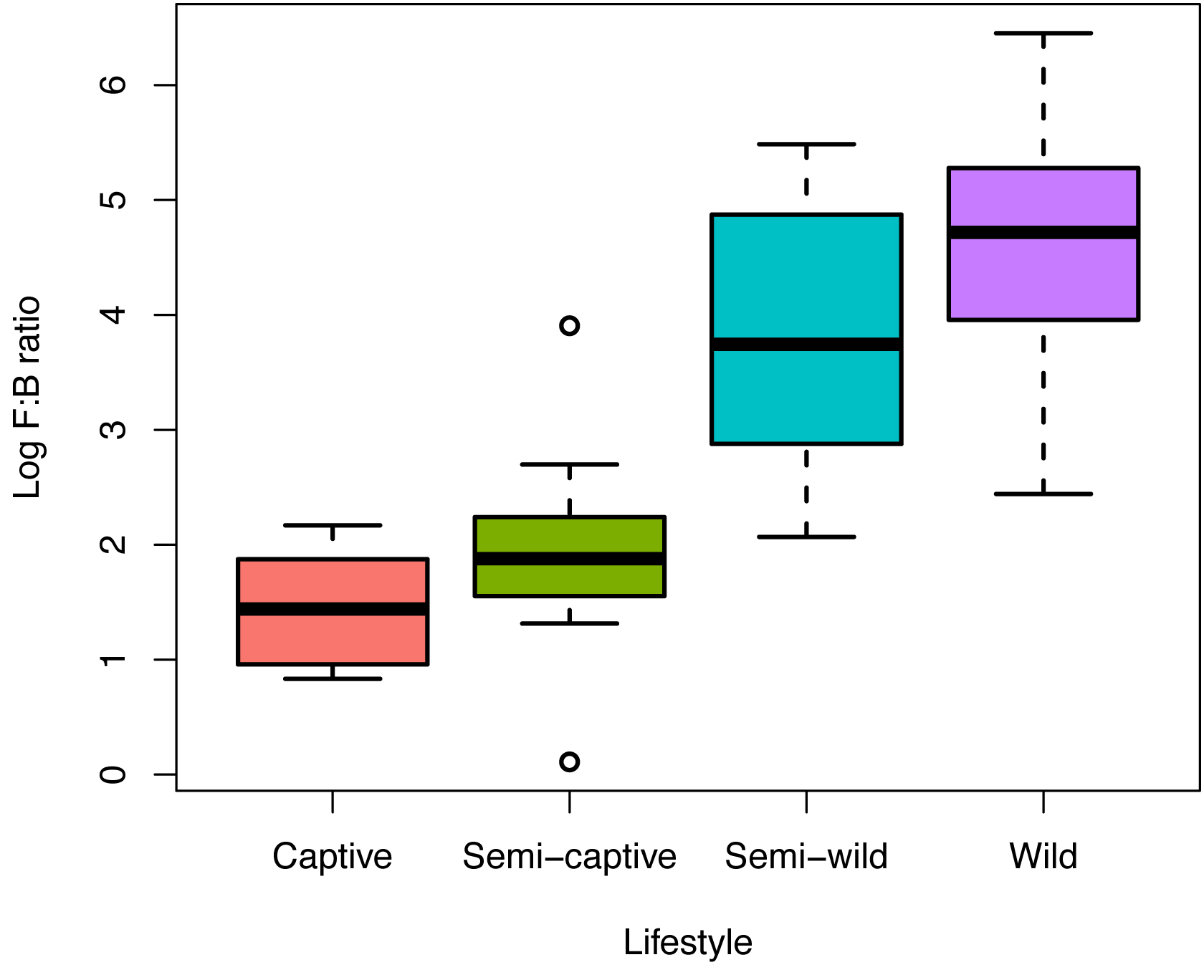


**Supplemental Figure 10. Firmicutes to Bacteroidetes ratio in red-shanked douc microbiomes across populations.** (a) Bar plots of *Firmicutes* to *Bacteroidetes* ratio, a measure of energy harvest capacity by microbial communities, plotted by wild, semi-wild, semi-captive, and captive populations of red-shanked doucs. All samples were obtained with the same protocol for V4 16S rRNA sequencing, and open-reference OTU picking was used. The *Firmicutes* to *Bacteroidetes* ratio was highest in wild doucs (4.64
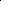
 0.94), followed by the semi-wild doucs (3.78
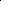
 1.14), semi-captive (1.94
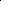
 0.81), and captive doucs (1.43
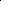
 0.50). A significant decrease in *Firmicutes* to *Bacteroidetes* ratio from the wild population to the semi-wild population, from semi-wild to the semi-captive population, and again from the semi-captive population to the captive population is visible. Pairwise Wilcoxon rank-sum p-values for the populations are: captive vs semi-captive p = 0.052858, captive vs semi-wild p = 4.62464e-08, captive vs wild p = 4.320356e-08, semi-captive vs semi-wild p = 5.177609e-06, semi-captive vs wild p = 6.900029e-09, semi-wild vs wild p = 0.006974937. (b) After collapsing by individual, the same decreasing diversity trend from wild to captive lifestyles is observed.

**SUPPLEMENTAL TABLES:**

**Supplemental Table 1: Differentially abundant genera.** (a) Statistical significance of differentiation was assessed using pairwise Wilcoxon rank-sum tests of each taxon’s centered-log-ratio transformed abundance in the combined captive & semi-captive lifestyle (n=27) versus wild lifestyle (n=66), as well as the polyserial correlation of the same across all four lifestyles as the ordered factor Wild < Semi-wild < Semi-captive < Captive. The polyserial correlation column is colored according to intensity of correlation; blue signifies decreased abundance with captivity level and yellow increased abundance with captivity level. Criteria for display included having Holm-adjusted Wilcoxon rank-sum p < 0.05, absolute polyserial correlation above 0.3, and polyserial rho p-value < 0.05. Taxa are displayed at the most specific taxonomic level to which they were annotated; taxa lacking annotation at the genus level can be interpreted as being “other” genera within the level of taxonomy they occupy. (b) This trend is robust to collapsing samples by individual (combined captive & semi-captive lifestyle n=9, wild lifestyle n=9; B-H adjusted p-values).

(a)

| **Taxa (up to Genus)** | **Polyserial Correlation** | **(Semi-)captive vs Wild Q** |
| --- | --- | --- |
| k__Bacteria;p__Firmicutes;c__Clostridia;o__Clostridiales;f__Lachnospiraceae;g__Shuttleworthia | -0.9699471 | 2.05E-09 |
| k__Bacteria;p__Proteobacteria;c__Alphaproteobacteria;o__Rickettsiales;f__Rickettsiaceae;g__Wolbachia | -0.8892253 | 0.000187015 |
| k__Bacteria;p__Actinobacteria;c__Coriobacteriia;o__Coriobacteriales;f__Coriobacteriaceae;g__Adlercreutzia | -0.8398788 | 1.02E-11 |
| k__Bacteria;p__Bacteroidetes;c__Bacteroidia;o__Bacteroidales;f__Bacteroidaceae | -0.8380668 | 1.01E-07 |
| k__Bacteria;p__Proteobacteria;c__Epsilonproteobacteria;o__Campylobacterales;f__Campylobacteraceae;g__Campylobacter | -0.8270076 | 2.99E-10 |
| k__Bacteria;p__Proteobacteria;c__Alphaproteobacteria;o__Rhodospirillales;f__Rhodospirillaceae | -0.825024 | 0.000439766 |
| k__Bacteria;p__Firmicutes;c__Bacilli;o__Bacillales;f__Planococcaceae | -0.8088798 | 0.000395901 |
| k__Bacteria;p__Firmicutes;c__Clostridia;o__Clostridiales;f__Lachnospiraceae;g__Clostridium | -0.8021905 | 0.0002493 |
| k__Bacteria;p__Proteobacteria;c__Alphaproteobacteria;o__Rhizobiales;f__Hyphomicrobiaceae;g__Rhodoplanes | -0.7650339 | 0.00050822 |
| k__Bacteria;p__Proteobacteria;c__Alphaproteobacteria;o__Rickettsiales;f__mitochondria | -0.761066 | 1.02E-11 |
| k__Bacteria;p__Tenericutes;c__Mollicutes;o__Anaeroplasmatales;f__Anaeroplasmataceae | -0.7606545 | 2.00E-11 |
| k__Bacteria;p__Firmicutes;c__Clostridia;o__Clostridiales;f__Lachnospiraceae;g__Dorea | -0.7495691 | 2.54E-08 |
| k__Bacteria;p__Proteobacteria;c__Alphaproteobacteria;o__Rhizobiales;f__Methylocystaceae | -0.7440362 | 0.000938513 |
| k__Bacteria;p__Firmicutes;c__Clostridia;o__Clostridiales;f__Dehalobacteriaceae;g__Dehalobacterium | -0.7394288 | 2.26E-11 |
| k__Bacteria;p__Firmicutes;c__Clostridia;o__Clostridiales;f__Ruminococcaceae;g__Oscillospira | -0.7392849 | 5.80E-11 |
| k__Bacteria;p__Firmicutes;c__Clostridia;o__Clostridiales;f__Lachnospiraceae;g__Blautia | -0.7340071 | 1.04E-09 |
| k__Bacteria;p__Verrucomicrobia;c__Opitutae;o__HA64 | -0.6889126 | 0.003672916 |
| k__Bacteria;p__Proteobacteria;c__Alphaproteobacteria;o__Rickettsiales | -0.6837371 | 0.000210592 |
| k__Bacteria;p__Firmicutes;c__Clostridia;o__Clostridiales;f__Veillonellaceae;g__Selenomonas | -0.6785613 | 0.0002493 |
| k__Bacteria;p__Actinobacteria;c__Actinobacteria;o__Actinomycetales;f__Frankiaceae | -0.6764886 | 0.00088488 |
| k__Bacteria;p__Firmicutes;c__Clostridia;o__Clostridiales;f__Veillonellaceae | -0.6748278 | 7.49E-06 |
| k__Bacteria;p__Firmicutes;c__Clostridia;o__Clostridiales;f__Lachnospiraceae;g__Anaerostipes | -0.651836 | 6.33E-07 |
| k__Bacteria;p__Proteobacteria;c__Alphaproteobacteria;o__Rhodospirillales;f__Acetobacteraceae | -0.6301408 | 0.00069679 |
| k__Bacteria;p__Proteobacteria;c__Betaproteobacteria;o__Burkholderiales;f__Comamonadaceae;g__Comamonas | -0.6292526 | 0.02536496 |
| k__Bacteria;p__Tenericutes;c__Mollicutes;o__RF39 | -0.625586 | 0.001970251 |
| k__Bacteria;p__Actinobacteria;c__Coriobacteriia;o__Coriobacteriales;f__Coriobacteriaceae | -0.6063056 | 8.99E-09 |
| k__Bacteria;p__Proteobacteria;c__Betaproteobacteria;o__Burkholderiales | -0.5887106 | 0.0118638 |
| k__Bacteria;p__Firmicutes;c__Clostridia;o__Clostridiales;f__Syntrophomonadaceae;g__Syntrophomonas | -0.5872707 | 0.006156004 |
| k__Bacteria;p__Firmicutes;c__Bacilli;o__Bacillales;f__Paenibacillaceae;g__Paenibacillus | -0.5866233 | 0.000439766 |
| k__Bacteria | -0.5848206 | 0.001918287 |
| k__Bacteria;p__Firmicutes;c__Clostridia;o__Clostridiales;f__Lachnospiraceae;g__Moryella | -0.577989 | 0.000216108 |
| k__Bacteria;p__Proteobacteria;c__Gammaproteobacteria;o__Xanthomonadales;f__Xanthomonadaceae | -0.5762689 | 0.009130425 |
| k__Bacteria;p__Verrucomicrobia;c__Verrucomicrobiae;o__Verrucomicrobiales;f__Verrucomicrobiaceae;g__Akkermansia | -0.5715996 | 1.29E-09 |
| k__Bacteria;p__Cyanobacteria;c__4C0d-2;o__YS2 | -0.5607493 | 0.000241094 |
| k__Bacteria;p__Tenericutes;c__RF3;o__ML615J-28 | -0.5542703 | 0.02536496 |
| k__Bacteria;p__Actinobacteria;c__Coriobacteriia;o__Coriobacteriales;f__Coriobacteriaceae;g__Atopobium | -0.5499778 | 9.63E-08 |
| k__Bacteria;p__Firmicutes;c__Clostridia;o__Clostridiales;f__EtOH8 | -0.5457671 | 0.001797864 |
| k__Bacteria;p__Firmicutes;c__Clostridia;o__Clostridiales;f__Lachnospiraceae;g__Lachnobacterium | -0.5430707 | 0.000156027 |
| k__Bacteria;p__Proteobacteria;c__Alphaproteobacteria;o__Rhizobiales;f__Bradyrhizobiaceae;g__Bradyrhizobium | -0.5269235 | 0.01718809 |
| k__Bacteria;p__Firmicutes;c__Clostridia;o__Clostridiales;f__Lachnospiraceae;g__Butyrivibrio | -0.517606 | 1.88E-07 |
| k__Bacteria;p__Firmicutes;c__Clostridia;o__Clostridiales;f__Lachnospiraceae;g__Coprococcus | -0.5076206 | 0.03173796 |
| k__Bacteria;p__Actinobacteria;c__Actinobacteria;o__Bifidobacteriales;f__Bifidobacteriaceae;g__Bifidobacterium | -0.506083 | 0.04068748 |
| k__Bacteria;p__Bacteroidetes;c__Bacteroidia;o__Bacteroidales;f__Porphyromonadaceae;g__Dysgonomonas | -0.5010785 | 0.012117 |
| k__Bacteria;p__Actinobacteria;c__Actinobacteria;o__Actinomycetales;f__Microbacteriaceae;g__Curtobacterium | -0.4990236 | 0.001183907 |
| k__Bacteria;p__Actinobacteria;c__Thermoleophilia;o__Solirubrobacterales | -0.4826565 | 0.001162568 |
| k__Bacteria;p__Proteobacteria;c__Alphaproteobacteria;o__Rhizobiales;f__Rhizobiaceae;g__Agrobacterium | -0.4681888 | 0.00017455 |
| k__Bacteria;p__Firmicutes;c__Clostridia;o__Clostridiales;f__Ruminococcaceae | -0.4419976 | 2.17E-08 |
| k__Bacteria;p__Firmicutes;c__Bacilli;o__Bacillales;f__Staphylococcaceae;g__Staphylococcus | -0.4289325 | 0.01752383 |
| k__Bacteria;p__Proteobacteria;c__Gammaproteobacteria;o__Pseudomonadales;f__Pseudomonadaceae | -0.3895052 | 0.02364246 |
| k__Bacteria;p__TM7;c__TM7-3;o__CW040;f__F16 | -0.3855686 | 0.000216108 |
| k__Bacteria;p__Proteobacteria;c__Alphaproteobacteria;o__Sphingomonadales;f__Sphingomonadaceae | -0.3203931 | 0.000911378 |
| k__Bacteria;p__Firmicutes;c__Bacilli;o__Bacillales | -0.3172278 | 0.001162568 |
| k__Bacteria;p__Proteobacteria;c__Alphaproteobacteria;o__Rhizobiales;f__Hyphomicrobiaceae;g__Devosia | -0.3157396 | 0.001004876 |
| k__Bacteria;p__Firmicutes;c__Erysipelotrichi;o__Erysipelotrichales;f__Erysipelotrichaceae;g__Bulleidia | 0.3813922 | 0.02450248 |
| k__Bacteria;p__Bacteroidetes;c__Bacteroidia;o__Bacteroidales;f__S24-7 | 0.4273285 | 1.13E-05 |
| k__Bacteria;p__Proteobacteria;c__Deltaproteobacteria;o__Desulfovibrionales;f__Desulfovibrionaceae | 0.4355352 | 8.98E-06 |
| k__Bacteria;p__Firmicutes;c__Clostridia;o__Clostridiales;f__Peptostreptococcaceae | 0.48779 | 0.01279911 |
| k__Bacteria;p__Verrucomicrobia;c__Verruco-5;o__WCHB1-41;f__RFP12 | 0.4960677 | 0.000255566 |
| k__Bacteria;p__Firmicutes;c__Clostridia;o__Clostridiales;f__Ruminococcaceae;g__Faecalibacterium | 0.5162926 | 3.22E-09 |
| k__Bacteria;p__Bacteroidetes;c__Bacteroidia;o__Bacteroidales;f__[Odoribacteraceae];g__Butyricimonas | 0.5359491 | 0.03027383 |
| k__Bacteria;p__Firmicutes;c__Bacilli;o__Lactobacillales;f__Lactobacillaceae;g__Lactobacillus | 0.5724962 | 0.000770276 |
| k__Bacteria;p__Bacteroidetes;c__Bacteroidia;o__Bacteroidales;f__p-2534-18B5;g__BE24 | 0.5802903 | 0.000425919 |
| k__Bacteria;p__Proteobacteria;c__Alphaproteobacteria;o__RF32 | 0.6515223 | 1.88E-07 |
| k__Bacteria;p__Proteobacteria;c__Deltaproteobacteria;o__GMD14H09 | 0.6669349 | 0.000568521 |
| k__Bacteria;p__Proteobacteria;c__Betaproteobacteria;o__Rhodocyclales;f__Rhodocyclaceae | 0.6705603 | 0.00262937 |
| k__Bacteria;p__Bacteroidetes;c__Bacteroidia;o__Bacteroidales;f__Rikenellaceae | 0.7092134 | 1.86E-10 |
| k__Bacteria;p__Bacteroidetes;c__Bacteroidia;o__Bacteroidales;f__[Paraprevotellaceae];g__YRC22 | 0.7118106 | 1.70E-06 |
| k__Bacteria;p__Spirochaetes;c__Spirochaetes;o__Spirochaetales;f__Spirochaetaceae;g__Treponema | 0.7119026 | 1.83E-09 |
| k__Bacteria;p__Lentisphaerae;c__[Lentisphaeria];o__Victivallales;f__Victivallaceae | 0.7404875 | 0.006156004 |
| k__Bacteria;p__Bacteroidetes;c__Bacteroidia;o__Bacteroidales;f__Prevotellaceae;g__Prevotella | 0.7438575 | 1.40E-08 |
| k__Bacteria;p__Bacteroidetes;c__Bacteroidia;o__Bacteroidales;f__RF16 | 0.7515577 | 0.01445429 |
| k__Bacteria;p__Bacteroidetes;c__Bacteroidia;o__Bacteroidales;f__[Paraprevotellaceae];g__CF231 | 0.7751741 | 6.04E-08 |
| k__Bacteria;p__Bacteroidetes;c__Bacteroidia;o__Bacteroidales | 0.7845308 | 6.06E-07 |
| k__Archaea;p__Euryarchaeota;c__Methanobacteria;o__Methanobacteriales;f__Methanobacteriaceae;g__Methanosphaera | 0.7990684 | 2.36E-10 |
| k__Bacteria;p__Bacteroidetes;c__Bacteroidia;o__Bacteroidales;f__Bacteroidaceae;g__Bacteroides | 0.8021878 | 1.14E-11 |

(b)

| Taxon (up to Genus) | Polyserial Correlation | (Semi-)captive vs Wild Q |
| --- | --- | --- |
| f__Lachnospiraceae;g__Shuttleworthia | -0.956483 | 0.000325343 |
| f__Rickettsiaceae;g__Wolbachia | -0.9185974 | 0.001073632 |
| f__Campylobacteraceae;g__Campylobacter | -0.9134617 | 0.000325343 |
| o__Bacillales;f__Planococcaceae | -0.9101858 | 0.005449497 |
| f__Clostridiaceae;g__Clostridium | -0.863446 | 0.000325343 |
| o__Rickettsiales;f__mitochondria | -0.8403265 | 0.000325343 |
| o__Bacteroidales;f__Bacteroidaceae | -0.8356222 | 0.000325343 |
| f__Coriobacteriaceae;g__Adlercreutzia | -0.7962237 | 0.000325343 |
| f__Hyphomicrobiaceae;g__Rhodoplanes | -0.7178556 | 0.000409003 |
| o__Anaeroplasmatales;f__Anaeroplasmataceae | -0.7045619 | 0.000325343 |
| f__Sphingomonadaceae;g__Sphingomonas | -0.6999661 | 0.001073632 |
| f__Lachnospiraceae;g__Blautia | -0.6830623 | 0.000409003 |
| c__Deltaproteobacteria;o__Myxococcales | -0.6803702 | 0.001073632 |
| f__Coriobacteriaceae;g__Atopobium | -0.6676546 | 0.000409003 |
| f__Dehalobacteriaceae;g__Dehalobacterium | -0.6230472 | 0.000325343 |
| p__Gemmatimonadetes;c__Gemm-1 | -0.6195835 | 0.001073632 |
| o__Coriobacteriales;f__Coriobacteriaceae | -0.6177747 | 0.000325343 |
| f__Verrucomicrobiaceae;g__Akkermansia | -0.614152 | 0.000325343 |
| f__Rhodocyclaceae;g__Dechloromonas | -0.6125506 | 0.001073632 |
| o__Clostridiales;f__Veillonellaceae | -0.6012192 | 0.01043175 |
| f__Lachnospiraceae;g__Lachnobacterium | -0.5998763 | 0.001638475 |
| f__Oxalobacteraceae;g__Oxalobacter | -0.5917583 | 0.001638475 |
| f__Lachnospiraceae;g__Dorea | -0.5873603 | 0.001073632 |
| f__[Chthoniobacteraceae];g__DA101 | -0.5823968 | 0.000409003 |
| f__Bifidobacteriaceae;g__Bifidobacterium | -0.5810479 | 0.000409003 |
| f__Ruminococcaceae;g__Oscillospira | -0.5805556 | 0.000325343 |
| f__Paenibacillaceae;g__Paenibacillus | -0.5791042 | 0.001073632 |
| f__Veillonellaceae;g__Selenomonas | -0.5786232 | 0.001073632 |
| o__Clostridiales;f__Lachnospiraceae | -0.5761906 | 0.001638475 |
| o__Rhodospirillales;f__Acetobacteraceae | -0.5683068 | 0.001073632 |
| k__Bacteria | -0.535979 | 0.001073632 |
| f__Xanthomonadaceae;g__Thermomonas | -0.535826 | 0.001073632 |
| c__Mollicutes;o__RF39 | -0.52672 | 0.1145208 |
| f__Lachnospiraceae;g__Butyrivibrio | -0.5247221 | 0.007629475 |
| f__Lachnospiraceae;g__Anaerostipes | -0.4966482 | 0.0305032 |
| f__Bradyrhizobiaceae;g__Bradyrhizobium | -0.4958424 | 0.000409003 |
| f__Lachnospiraceae;g__Moryella | -0.4917757 | 0.01409142 |
| o__Actinomycetales;f__Nocardioidaceae | -0.4880457 | 0.001073632 |
| c__4C0d-2;o__YS2 | -0.485183 | 0.07678668 |
| o__Rhizobiales;f__Beijerinckiaceae | -0.4700109 | 0.001073632 |
| f__Erysipelotrichaceae;g__Sharpea | -0.455839 | 0.01828364 |
| o__Clostridiales;f__[Mogibacteriaceae] | -0.439081 | 0.003745229 |
| o__Actinomycetales;f__Micrococcaceae | -0.4240343 | 0.001073632 |
| o__RB41;f__Ellin6075 | -0.4207194 | 0.001073632 |
| c__Clostridia;o__Clostridiales | -0.4197319 | 0.4606324 |
| c__Alphaproteobacteria;o__Rickettsiales | -0.4178271 | 0.2128828 |
| f__Porphyromonadaceae;g__Dysgonomonas | -0.4154784 | 0.01828364 |
| f__Syntrophomonadaceae;g__Syntrophomonas | -0.414149 | 0.01043175 |
| f__Carnobacteriaceae;g__Granulicatella | -0.4060651 | 0.000409003 |
| c__Bacilli;o__Bacillales | -0.3994767 | 0.000409003 |
| f__Moraxellaceae;g__Acinetobacter | -0.3848474 | 0.07678668 |
| o__Clostridiales;f__Clostridiaceae | -0.3815454 | 0.002556267 |
| f__[Tissierellaceae];g__Anaerococcus | -0.3713166 | 0.001073632 |
| c__RF3;o__ML615J-28 | -0.3681541 | 0.323068 |
| f__Gemellaceae;g__Gemella | -0.3653648 | 0.01828364 |
| f__Corynebacteriaceae;g__Corynebacterium | -0.3613869 | 0.001073632 |
| o__Actinomycetales;f__Microbacteriaceae | -0.3565785 | 0.001073632 |
| f__Hyphomicrobiaceae;g__Devosia | -0.3520108 | 0.001073632 |
| o__Clostridiales;f__Ruminococcaceae | -0.3513601 | 0.000325343 |
| o__Sphingomonadales;f__Sphingomonadaceae | -0.3408164 | 0.001073632 |
| f__Dermabacteraceae;g__Brachybacterium | -0.3401626 | 0.001073632 |
| o__CW040;f__F16 | -0.3277161 | 0.001073632 |
| f__Rhizobiaceae;g__Agrobacterium | -0.3260375 | 0.001073632 |
| c__Bacilli;o__Lactobacillales | -0.3237024 | 0.000409003 |
| o__Bacteroidales;f__[Barnesiellaceae] | -0.321496 | 0.03936652 |
| f__Enterococcaceae;g__Enterococcus | -0.3168855 | 0.000409003 |
| o__Sphingomonadales;f__Erythrobacteraceae | -0.3159322 | 0.001073632 |
| o__Pirellulales;f__Pirellulaceae | 0.3690673 | 0.5060864 |
| o__Rhodocyclales;f__Rhodocyclaceae | 0.3828332 | 0.5060864 |
| o__Bifidobacteriales;f__Bifidobacteriaceae | 0.3995816 | 0.323068 |
| o__WCHB1-41;f__RFP12 | 0.4305388 | 0.60479 |
| f__Erysipelotrichaceae;g__p-75-a5 | 0.4361823 | 0.1554935 |
| o__Z20;f__R4-45B | 0.4785572 | 0.1554935 |
| o__Campylobacterales;f__Helicobacteraceae | 0.483107 | 0.1554935 |
| f__Lactobacillaceae;g__Lactobacillus | 0.4833548 | 0.000325343 |
| o__Bacteroidales;f__S24-7 | 0.5060037 | 0.003745229 |
| o__Elusimicrobiales;f__Elusimicrobiaceae | 0.5237921 | 0.4606324 |
| c__Deltaproteobacteria;o__GMD14H09 | 0.5647573 | 0.7071134 |
| f__[Mogibacteriaceae];g__Mogibacterium | 0.5805199 | 0.05023417 |
| c__Alphaproteobacteria;o__RF32 | 0.5837384 | 0.1847542 |
| f__[Paraprevotellaceae];g__[Prevotella] | 0.61268 | 0.1554935 |
| o__Bacteroidales;f__RF16 | 0.6186098 | 0.7071134 |
| f__Ruminococcaceae;g__Faecalibacterium | 0.6256625 | 0.000325343 |
| f__p-2534-18B5;g__BE24 | 0.6275738 | 0.2128828 |
| f__Prevotellaceae;g__Prevotella | 0.6399604 | 0.001073632 |
| o__Bacteroidales;f__BS11 | 0.6466554 | 0.829547 |
| f__Veillonellaceae;g__Anaerovibrio | 0.6549306 | 0.9368114 |
| o__Bacteroidales;f__Rikenellaceae | 0.6677913 | 0.000325343 |
| f__[Paraprevotellaceae];g__YRC22 | 0.678498 | 0.06267691 |
| f__Veillonellaceae;g__Phascolarctobacterium | 0.6829802 | 0.000325343 |
| f__Desulfovibrionaceae;g__Desulfovibrio | 0.6857732 | 0.000325343 |
| c__Bacteroidia;o__Bacteroidales | 0.7080042 | 0.06267691 |
| o__Victivallales;f__Victivallaceae | 0.7082786 | 0.5602665 |
| f__Methanobacteriaceae;g__Methanobrevibacter | 0.7329222 | 0.000325343 |
| f__Methanobacteriaceae;g__Methanosphaera | 0.7392959 | 0.005449497 |
| f__[Paraprevotellaceae];g__CF231 | 0.7471837 | 0.007629475 |
| f__Spirochaetaceae;g__Treponema | 0.7785781 | 0.000325343 |
| f__Bacteroidaceae;g__Bacteroides | 0.865261 | 0.000325343 |
| f__Porphyromonadaceae;g__Parabacteroides | 0.9360479 | 0.000325343 |

**Supplemental Table 2. Differentially abundant functional pathways.** (a) Statistical significance of differentiation was assessed using pairwise Wilcoxon rank-sum tests of each pathway’s centered-log-ratio transformed abundance between a combined captive & semi-captive lifestyle (n=27) versus wild lifestyle (n=66), as well as the polyserial correlation of each pathway across all four lifestyles as the ordered factor Wild < Semi-wild < Semi-captive < Captive. The polyserial correlation column is colored according to intensity of correlation; blue signifies decreased abundance with captivity level and yellow increased abundance with captivity level. Criteria for display included having Holm-adjusted Wilcoxon rank-sum p < 0.05, absolute polyserial correlation above 0.3, and polyserial rho p-value < 0.05. (b) This trend is robust to collapsing samples by individual (combined captive & semi-captive lifestyle n=9, wild lifestyle n=9; B-H adjusted p-values).

(a)

| **Pathway** | **Polyserial Correlation** | **(Semi-)captive vs Wild Q** |
| --- | --- | --- |
| Ubiquinone and other terpenoid-quinone biosynthesis | 0.86613 | 1.65E-11 |
| Vibrio cholerae pathogenic cycle | 0.8522796 | 1.76E-10 |
| Amino acid metabolism | 0.8485574 | 2.21E-11 |
| Toluene degradation | 0.8355032 | 2.63E-11 |
| Lipoic acid metabolism | 0.8349192 | 1.47E-11 |
| Proximal tubule bicarbonate reclamation | 0.8285716 | 2.34E-11 |
| Peroxisome | 0.8264319 | 2.34E-11 |
| D-Glutamine and D-glutamate metabolism | 0.809642 | 1.32E-11 |
| Transcription related proteins | 0.8057511 | 1.95E-11 |
| Translation factors | 0.8016383 | 1.40E-11 |
| Protein processing in endoplasmic reticulum | 0.7868988 | 2.62E-10 |
| Inositol phosphate metabolism | 0.7778543 | 1.86E-10 |
| Vitamin B6 metabolism | 0.7715443 | 2.96E-11 |
| Base excision repair | 0.7714182 | 2.92E-10 |
| Biosynthesis of vancomycin group antibiotics | 0.7689167 | 2.76E-10 |
| Inorganic ion transport and metabolism | 0.7637347 | 1.60E-10 |
| Glycine, serine and threonine metabolism | 0.7581197 | 7.85E-10 |
| Oxidative phosphorylation | 0.7568452 | 6.65E-10 |
| beta-Lactam resistance | 0.7432696 | 1.91E-09 |
| Phosphatidylinositol signaling system | 0.739012 | 6.11E-10 |
| Isoflavonoid biosynthesis | 0.7284541 | 2.31E-10 |
| Alanine, aspartate and glutamate metabolism | 0.7259672 | 3.26E-09 |
| Tyrosine metabolism | 0.7253912 | 2.92E-10 |
| Purine metabolism | 0.7253711 | 8.19E-09 |
| Metabolism of cofactors and vitamins | 0.7236583 | 6.63E-09 |
| Function unknown | 0.7168606 | 6.63E-09 |
| Ascorbate and aldarate metabolism | 0.7161581 | 5.06E-09 |
| Protein folding and associated processing | 0.7070021 | 3.26E-09 |
| Glutamatergic synapse | 0.7026025 | 4.82E-11 |
| Ubiquitin system | 0.7021036 | 9.09E-11 |
| One carbon pool by folate | 0.694698 | 3.15E-11 |
| General function prediction only | 0.6884648 | 2.14E-08 |
| Polyketide sugar unit biosynthesis | 0.685304 | 1.32E-08 |
| Nucleotide excision repair | 0.6826238 | 1.92E-08 |
| Other ion-coupled transporters | 0.677045 | 8.62E-09 |
| Phosphonate and phosphinate metabolism | 0.6754849 | 1.86E-10 |
| Sphingolipid metabolism | 0.6597281 | 6.30E-09 |
| Valine, leucine and isoleucine degradation | 0.6583393 | 1.07E-10 |
| Aminobenzoate degradation | 0.6487151 | 1.68E-10 |
| Renal cell carcinoma | 0.6353665 | 3.17E-08 |
| Styrene degradation | 0.6319073 | 5.33E-09 |
| Retinol metabolism | 0.6318299 | 1.02E-07 |
| Lysine degradation | 0.6300815 | 1.68E-10 |
| Ethylbenzene degradation | 0.627926 | 4.13E-07 |
| beta-Alanine metabolism | 0.6127058 | 1.84E-08 |
| Huntington's disease | 0.6073938 | 7.66E-10 |
| Primary immunodeficiency | 0.5987362 | 2.05E-06 |
| Bacterial secretion system | 0.5980548 | 1.57E-06 |
| Tryptophan metabolism | 0.5884344 | 4.71E-10 |
| Carbohydrate digestion and absorption | 0.5876023 | 5.04E-05 |
| Glyoxylate and dicarboxylate metabolism | 0.58556 | 1.76E-07 |
| Geraniol degradation | 0.5758705 | 3.44E-08 |
| Pentose and glucuronate interconversions | 0.574738 | 2.94E-06 |
| Proteasome | 0.5693384 | 1.14E-09 |
| Glutathione metabolism | 0.5680232 | 2.93E-09 |
| Primary bile acid biosynthesis | 0.5644047 | 1.90E-05 |
| Bacterial toxins | 0.5640835 | 3.57E-07 |
| DNA repair and recombination proteins | 0.5619595 | 6.34E-07 |
| Butanoate metabolism | 0.5611606 | 1.43E-05 |
| Sulfur metabolism | 0.5512656 | 9.22E-06 |
| Carbon fixation in photosynthetic organisms | 0.5487902 | 7.47E-05 |
| Cell cycle - Caulobacter | 0.5453256 | 6.84E-06 |
| Influenza A | 0.5451721 | 3.07E-09 |
| Arginine and proline metabolism | 0.5397563 | 4.52E-07 |
| Renin-angiotensin system | 0.5395988 | 8.61E-05 |
| Peptidases | 0.5366534 | 2.69E-06 |
| Secondary bile acid biosynthesis | 0.5348466 | 0.000119449 |
| Pathways in cancer | 0.5310567 | 1.59E-07 |
| Porphyrin and chlorophyll metabolism | 0.5304321 | 2.36E-06 |
| Penicillin and cephalosporin biosynthesis | 0.5278086 | 1.32E-07 |
| Epithelial cell signaling in Helicobacter pylori infection | 0.5246982 | 2.58E-08 |
| Taurine and hypotaurine metabolism | 0.5205521 | 2.64E-06 |
| Fatty acid metabolism | 0.5196525 | 7.33E-07 |
| Linoleic acid metabolism | 0.514908 | 2.21E-05 |
| Limonene and pinene degradation | 0.5124961 | 2.25E-07 |
| Pyruvate metabolism | 0.5080889 | 8.66E-06 |
| Betalain biosynthesis | 0.5051249 | 1.12E-07 |
| Caprolactam degradation | 0.5033751 | 1.84E-08 |
| Bladder cancer | 0.5029604 | 2.16E-07 |
| Arachidonic acid metabolism | 0.4984154 | 0.03065246 |
| Indole alkaloid biosynthesis | 0.4961972 | 8.62E-09 |
| Chlorocyclohexane and chlorobenzene degradation | 0.4911376 | 1.48E-07 |
| Adipocytokine signaling pathway | 0.4900165 | 4.52E-07 |
| Meiosis - yeast | 0.4840796 | 4.69E-07 |
| Peptidoglycan biosynthesis | 0.4716894 | 8.99E-08 |
| Hypertrophic cardiomyopathy (HCM) | 0.4646138 | 4.78E-06 |
| Glycosphingolipid biosynthesis - lacto and neolacto series | 0.463966 | 6.35E-06 |
| Bisphenol degradation | 0.4605462 | 0.000143559 |
| Apoptosis | 0.4585675 | 6.01E-08 |
| Amyotrophic lateral sclerosis (ALS) | 0.4543189 | 8.12E-06 |
| Metabolism of xenobiotics by cytochrome P450 | 0.4531665 | 1.56E-05 |
| Drug metabolism - cytochrome P450 | 0.4502912 | 9.81E-06 |
| Circadian rhythm - plant | 0.4451969 | 0.000116798 |
| Naphthalene degradation | 0.4430097 | 0.002843726 |
| Amino acid related enzymes | 0.4348306 | 0.000212966 |
| Protein export | 0.4147701 | 0.000265293 |
| Propanoate metabolism | 0.4059516 | 0.002464333 |
| Amino sugar and nucleotide sugar metabolism | 0.3813197 | 0.004732689 |
| Selenocompound metabolism | 0.3793765 | 0.007380699 |
| Tuberculosis | 0.3765017 | 0.000983163 |
| Stilbenoid, diarylheptanoid and gingerol biosynthesis | 0.3715535 | 0.00017531 |
| Biosynthesis of unsaturated fatty acids | 0.364953 | 0.006819423 |
| Fluorobenzoate degradation | 0.3606798 | 0.003021935 |
| Synthesis and degradation of ketone bodies | 0.3599332 | 0.008969831 |
| Galactose metabolism | 0.3505926 | 0.02778041 |
| Cyanoamino acid metabolism | 0.350403 | 0.01341671 |
| Ether lipid metabolism | 0.346602 | 0.004607714 |
| p53 signaling pathway | 0.3424512 | 0.001598621 |
| Colorectal cancer | 0.3389656 | 0.002464333 |
| Small cell lung cancer | 0.3389656 | 0.002464333 |
| Toxoplasmosis | 0.3389656 | 0.002464333 |
| Viral myocarditis | 0.3389656 | 0.002464333 |
| Flavone and flavonol biosynthesis | 0.3362552 | 0.03059393 |
| D-Alanine metabolism | 0.3099026 | 0.04758065 |
| Flagellar assembly | -0.4501591 | 0.01617119 |
| Restriction enzyme | -0.4623062 | 6.60E-05 |
| Bacterial chemotaxis | -0.472489 | 0.006819423 |
| Bacterial invasion of epithelial cells | -0.5033562 | 2.75E-08 |
| Calcium signaling pathway | -0.5058255 | 1.74E-10 |
| Photosynthesis - antenna proteins | -0.5096998 | 1.60E-10 |
| Dioxin degradation | -0.5236634 | 0.000565156 |
| Chloroalkane and chloroalkene degradation | -0.5241747 | 0.000413393 |
| Bacterial motility proteins | -0.5446488 | 0.000532686 |
| Replication, recombination and repair proteins | -0.5446959 | 0.000426365 |
| Tetracycline biosynthesis | -0.5466325 | 0.000769446 |
| Xylene degradation | -0.6628035 | 2.05E-07 |
| Mineral absorption | -0.6630577 | 4.53E-09 |
| Sporulation | -0.7151384 | 1.23E-09 |
| African trypanosomiasis | -0.7423802 | 1.42E-10 |
| Germination | -0.7761985 | 6.15E-11 |
| Atrazine degradation | -0.8109147 | 2.62E-10 |
| Cytoskeleton proteins | -0.824936 | 1.86E-10 |
| Lipid metabolism | -0.8911117 | 1.40E-11 |

(b)

| **Pathway** | **Polyserial Correlation** | **(Semi-)captive vs Wild Q** |
| --- | --- | --- |
| Isoquinoline alkaloid biosynthesis | 0.962452 | 0.000110626 |
| Protein digestion and absorption | 0.9318032 | 0.000110626 |
| Amino acid metabolism | 0.9103115 | 0.000110626 |
| Cell division | 0.8993256 | 0.000110626 |
| Energy metabolism | 0.8929383 | 0.000110626 |
| Lipopolysaccharide biosynthesis | 0.8896145 | 0.000110626 |
| N-Glycan biosynthesis | 0.8886765 | 0.000110626 |
| Zeatin biosynthesis | 0.88831 | 0.000110626 |
| Cellular antigens | 0.8880107 | 0.000110626 |
| Nicotinate and nicotinamide metabolism | 0.8847374 | 0.000110626 |
| Glycosphingolipid biosynthesis - ganglio series | 0.8808071 | 0.000110626 |
| Lipopolysaccharide biosynthesis proteins | 0.8799688 | 0.000110626 |
| Tropane, piperidine and pyridine alkaloid biosynthesis | 0.8793397 | 0.000110626 |
| Bile secretion | 0.878801 | 0.000872465 |
| Prenyltransferases | 0.8725163 | 0.000110626 |
| Glycosaminoglycan degradation | 0.8672038 | 0.000110626 |
| Membrane and intracellular structural molecules | 0.8670879 | 0.000110626 |
| Proximal tubule bicarbonate reclamation | 0.8462325 | 0.000110626 |
| Pertussis | 0.843898 | 0.000110626 |
| Vibrio cholerae pathogenic cycle | 0.8425944 | 0.000110626 |
| Pyrimidine metabolism | 0.8404925 | 0.000110626 |
| Novobiocin biosynthesis | 0.8398676 | 0.000110626 |
| Lysosome | 0.8386217 | 0.000110626 |
| Pores ion channels | 0.8278502 | 0.000110626 |
| Citrate cycle (TCA cycle) | 0.8264482 | 0.000110626 |
| Cell motility and secretion | 0.8247574 | 0.000110626 |
| Folate biosynthesis | 0.8202202 | 0.000110626 |
| Basal transcription factors | 0.8184619 | 0.000110626 |
| Peroxisome | 0.8076425 | 0.000110626 |
| Methane metabolism | 0.8074814 | 0.000110626 |
| Various types of N-glycan biosynthesis | 0.8043196 | 0.000872465 |
| Vibrio cholerae infection | 0.8025811 | 0.000110626 |
| Drug metabolism - other enzymes | 0.8022127 | 0.000110626 |
| Cell cycle | 0.7995205 | 0.000110626 |
| Hepatitis C | 0.7995205 | 0.000110626 |
| Measles | 0.7995205 | 0.000110626 |
| Phagosome | 0.7995205 | 0.000110626 |
| mTOR signaling pathway | 0.7995205 | 0.000110626 |
| mRNA surveillance pathway | 0.797594 | 0.000110626 |
| Ribosome biogenesis in eukaryotes | 0.7937459 | 0.000110626 |
| D-Glutamine and D-glutamate metabolism | 0.7880353 | 0.000110626 |
| RNA degradation | 0.7851257 | 0.000110626 |
| Glycine, serine and threonine metabolism | 0.7813736 | 0.000110626 |
| Other glycan degradation | 0.7794628 | 0.000110626 |
| Steroid hormone biosynthesis | 0.7762508 | 0.000110626 |
| Other ion-coupled transporters | 0.7681835 | 0.000110626 |
| Lipoic acid metabolism | 0.7631028 | 0.000110626 |
| Glycosphingolipid biosynthesis - globo series | 0.7628271 | 0.000110626 |
| Streptomycin biosynthesis | 0.7538768 | 0.000110626 |
| Chaperones and folding catalysts | 0.7536326 | 0.000110626 |
| Translation factors | 0.7495534 | 0.000110626 |
| General function prediction only | 0.7451157 | 0.000110626 |
| Carbon fixation pathways in prokaryotes | 0.7446021 | 0.000110626 |
| Ubiquinone and other terpenoid-quinone biosynthesis | 0.7384093 | 0.000110626 |
| Alanine, aspartate and glutamate metabolism | 0.734631 | 0.000110626 |
| Purine metabolism | 0.731486 | 0.000110626 |
| Histidine metabolism | 0.7313133 | 0.000110626 |
| Base excision repair | 0.7249521 | 0.000110626 |
| Biotin metabolism | 0.7206835 | 0.000211009 |
| Phenylalanine metabolism | 0.7178694 | 0.000110626 |
| Glutamatergic synapse | 0.7174346 | 0.000110626 |
| Oxidative phosphorylation | 0.7169988 | 0.000110626 |
| MAPK signaling pathway - yeast | 0.714121 | 0.000110626 |
| One carbon pool by folate | 0.7135504 | 0.000110626 |
| Vitamin B6 metabolism | 0.7110365 | 0.000110626 |
| Pentose and glucuronate interconversions | 0.7067016 | 0.000211009 |
| Butirosin and neomycin biosynthesis | 0.7036575 | 0.000110626 |
| Bacterial toxins | 0.7026107 | 0.000110626 |
| Biosynthesis of vancomycin group antibiotics | 0.7011543 | 0.000110626 |
| Other transporters | 0.699843 | 0.000110626 |
| beta-Lactam resistance | 0.6920856 | 0.000110626 |
| Polyketide sugar unit biosynthesis | 0.6920504 | 0.000110626 |
| Phosphonate and phosphinate metabolism | 0.6914129 | 0.000110626 |
| Toluene degradation | 0.6887733 | 0.000110626 |
| Linoleic acid metabolism | 0.6877623 | 0.000389555 |
| Sphingolipid metabolism | 0.6856119 | 0.000110626 |
| DNA repair and recombination proteins | 0.6726807 | 0.000110626 |
| Inositol phosphate metabolism | 0.6696865 | 0.000110626 |
| Peptidases | 0.6668799 | 0.000110626 |
| Carbon fixation in photosynthetic organisms | 0.6474575 | 0.000389555 |
| Ascorbate and aldarate metabolism | 0.6447857 | 0.000110626 |
| Protein processing in endoplasmic reticulum | 0.638072 | 0.000389555 |
| Inorganic ion transport and metabolism | 0.6332071 | 0.000110626 |
| Isoflavonoid biosynthesis | 0.6323306 | 0.001113363 |
| Porphyrin and chlorophyll metabolism | 0.627808 | 0.000110626 |
| Primary bile acid biosynthesis | 0.6215682 | 0.001453917 |
| Terpenoid backbone biosynthesis | 0.6140327 | 0.000110626 |
| Cell cycle - Caulobacter | 0.6108282 | 0.000110626 |
| Transcription related proteins | 0.6100168 | 0.000110626 |
| Secondary bile acid biosynthesis | 0.6054719 | 0.000638091 |
| Primary immunodeficiency | 0.601115 | 0.000211009 |
| Arginine and proline metabolism | 0.591485 | 0.000211009 |
| Function unknown | 0.5866045 | 0.000110626 |
| Sulfur metabolism | 0.5856944 | 0.000389555 |
| Epithelial cell signaling in Helicobacter pylori infection | 0.5826354 | 0.000110626 |
| Glycosyltransferases | 0.5816984 | 0.001452988 |
| Nucleotide excision repair | 0.579885 | 0.000110626 |
| Ubiquitin system | 0.5785945 | 0.000872465 |
| Amino sugar and nucleotide sugar metabolism | 0.5767257 | 0.002191248 |
| Protein folding and associated processing | 0.5713211 | 0.000110626 |
| Riboflavin metabolism | 0.5693848 | 0.000990825 |
| Bisphenol degradation | 0.5672808 | 0.000638091 |
| Pyruvate metabolism | 0.565221 | 0.000389555 |
| Cysteine and methionine metabolism | 0.5567748 | 0.000110626 |
| Metabolism of cofactors and vitamins | 0.5485568 | 0.001452988 |
| Protein export | 0.5460609 | 0.001452988 |
| Type I diabetes mellitus | 0.5403533 | 0.000389555 |
| Tyrosine metabolism | 0.5368632 | 0.000110626 |
| Butanoate metabolism | 0.5345861 | 0.000638091 |
| Ethylbenzene degradation | 0.5316364 | 0.000211009 |
| Glyoxylate and dicarboxylate metabolism | 0.5295469 | 0.000110626 |
| Phenylalanine, tyrosine and tryptophan biosynthesis | 0.5240678 | 0.000990825 |
| D-Arginine and D-ornithine metabolism | 0.5228124 | 0.001452988 |
| Proteasome | 0.5167182 | 0.000110626 |
| Styrene degradation | 0.5158974 | 0.000110626 |
| Cyanoamino acid metabolism | 0.5033649 | 0.000990825 |
| Selenocompound metabolism | 0.4965108 | 0.000990825 |
| Galactose metabolism | 0.4907949 | 0.003165136 |
| Amino acid related enzymes | 0.4875685 | 0.000389555 |
| Phosphatidylinositol signaling system | 0.4775303 | 0.002191248 |
| DNA replication proteins | 0.4740188 | 0.004438551 |
| Aminobenzoate degradation | 0.4597159 | 0.000110626 |
| Valine, leucine and isoleucine degradation | 0.4592018 | 0.000110626 |
| Lysine degradation | 0.4577376 | 0.000110626 |
| Peptidoglycan biosynthesis | 0.4504085 | 0.000638091 |
| Biosynthesis and biodegradation of secondary metabolites | 0.4432297 | 0.004438551 |
| Fructose and mannose metabolism | 0.4424148 | 0.02026339 |
| Renal cell carcinoma | 0.4338874 | 0.001452988 |
| Penicillin and cephalosporin biosynthesis | 0.4290964 | 0.003165136 |
| beta-Alanine metabolism | 0.4199385 | 0.002191248 |
| Taurine and hypotaurine metabolism | 0.4088479 | 0.002191248 |
| Huntington's disease | 0.4073318 | 0.000110626 |
| Adipocytokine signaling pathway | 0.3889667 | 0.008577169 |
| Ribosome | 0.3834054 | 0.1428972 |
| Flavone and flavonol biosynthesis | 0.3775579 | 0.002191248 |
| Tryptophan metabolism | 0.3766433 | 0.000110626 |
| Pathways in cancer | 0.3742303 | 0.000638091 |
| Endocytosis | 0.3712485 | 0.004073343 |
| Fc gamma R-mediated phagocytosis | 0.3712485 | 0.004073343 |
| GnRH signaling pathway | 0.3712485 | 0.004073343 |
| C5-Branched dibasic acid metabolism | 0.3689664 | 0.01151635 |
| Glycerophospholipid metabolism | 0.3670306 | 0.01547252 |
| Glycosphingolipid biosynthesis - lacto and neolacto series | 0.3664238 | 0.00221204 |
| Geraniol degradation | 0.3609707 | 0.000638091 |
| Glycan biosynthesis and metabolism | 0.3597479 | 0.1006155 |
| Phenylpropanoid biosynthesis | 0.3568653 | 0.01151635 |
| PPAR signaling pathway | 0.3561933 | 0.01547252 |
| Lysine biosynthesis | 0.3539641 | 0.008577169 |
| Bacterial secretion system | 0.3378971 | 0.008577169 |
| Translation proteins | 0.3373794 | 0.01151635 |
| Tuberculosis | 0.3272165 | 0.003165136 |
| Hypertrophic cardiomyopathy (HCM) | 0.3212134 | 0.01922124 |
| Indole alkaloid biosynthesis | 0.3137979 | 0.00555021 |
| Transcription machinery | 0.3130225 | 0.001452988 |
| Glutathione metabolism | 0.3093002 | 0.000990825 |
| Pentose phosphate pathway | 0.3092835 | 0.2577824 |
| Retinol metabolism | 0.3074146 | 0.01151635 |
| D-Alanine metabolism | 0.3050444 | 0.082869 |
| RNA transport | 0.3027676 | 0.1428972 |
| Fatty acid metabolism | 0.3024075 | 0.001452988 |
| Bacterial chemotaxis | -0.3073898 | 0.082869 |
| Alzheimer's disease | -0.3351378 | 0.000990825 |
| Staphylococcus aureus infection | -0.346025 | 0.000110626 |
| Biosynthesis of siderophore group nonribosomal peptides | -0.3678083 | 0.000990825 |
| Restriction enzyme | -0.3967474 | 0.000389555 |
| Secretion system | -0.4225848 | 0.02026339 |
| Replication, recombination and repair proteins | -0.4226466 | 0.004438551 |
| Dioxin degradation | -0.4303023 | 0.008577169 |
| RNA polymerase | -0.4393173 | 0.004438551 |
| Chloroalkane and chloroalkene degradation | -0.445368 | 0.006279917 |
| Flagellar assembly | -0.4455676 | 0.04315239 |
| Tetracycline biosynthesis | -0.4645253 | 0.02650962 |
| Sporulation | -0.4646379 | 0.000638091 |
| Plant-pathogen interaction | -0.4806699 | 0.006279917 |
| Xylene degradation | -0.5052916 | 0.005653769 |
| Bacterial motility proteins | -0.5070289 | 0.01547252 |
| Bacterial invasion of epithelial cells | -0.5657188 | 0.002982558 |
| Calcium signaling pathway | -0.6016257 | 0.000872465 |
| Photosynthesis - antenna proteins | -0.60684 | 0.000110626 |
| Cytoskeleton proteins | -0.6609158 | 0.000110626 |
| Germination | -0.6656536 | 0.000110626 |
| Mineral absorption | -0.6672607 | 0.000110626 |
| Atrazine degradation | -0.8157228 | 0.000110626 |
| African trypanosomiasis | -0.8371311 | 0.000110626 |
| Aminoacyl-tRNA biosynthesis | -0.8419617 | 0.000110626 |
| Biosynthesis of 12-, 14- and 16-membered macrolides | -0.8424975 | 0.2037577 |
| Prion diseases | -0.8449049 | 0.000872465 |
| Lipid metabolism | -0.8569292 | 0.000110626 |
| Biosynthesis of type II polyketide backbone | -0.9830079 | 0.2037577 |

**Supplemental Table 3: Plant orders observed in douc populations by lifestyle.**

| **Diets by Lifestyle** | **Captive** | **Semi-captive** | **Semi-wild** | **Wild** |
| --- | --- | --- | --- | --- |
| Plant order/orders | Rosales | *Brassicales  Caryophyllales  *Fabales  Lamiales  Laurales  Malpighiales  Malvales  *Myrtales  Rosales  Sapindales | Aquifoliales  *Asterales  *Brassicales  *Cornales  *Ericales  *Fabales  *Gentianales  *Lamiales  *Laurales  *Malpighiales  *Malvales  Oxalidales  *Rosales  *Sapindales | Apiales  Aquifoliales  *Caryophyllales  *Ericales  *Fabales  *Fagales  *Gentianales  *Lamiales  *Laurales  Magnoliales  *Malpighiales  *Malvales  *Myrtales  *Rosales  *Sapindales |

*Plant orders recovered via 16S rRNA sequencing (at least 5 sequences observed in the lifestyle)
